# Amplifier Hosts May Play an Essential Role in Tomato Begomovirus Epidemics in Brazil

**DOI:** 10.3389/fpls.2020.00414

**Published:** 2020-04-15

**Authors:** Armando Bergamin Filho, Mônica A. Macedo, Gabriel M. Favara, Daiana Bampi, de Felipe F. Oliveira, Jorge A. M. Rezende

**Affiliations:** Department of Plant Pathology and Nematology, E.S.A. “Luiz de Queiroz,” University of São Paulo, Piracicaba, Brazil

**Keywords:** *Solanum lycopersicum*, *Bemisia tabaci*, Geminiviridae, reservoir, epidemiology

## Abstract

Current control of tomato golden mosaic disease, caused in Brazil predominantly by tomato severe rugose virus (ToSRV), is dependent on both, planting resistant/tolerant hybrids and intensive insecticide sprays (two to three per week) for controlling *Bemisia tabaci*, the vector of ToSRV. Resistant hybrids only confer moderate resistance to infection by ToSRV and some tolerance to the disease. Insecticide sprays, although widely used, have failed in most tomato production areas in Brazil, as they are unable to reduce primary spread, i.e., infection caused by the influx of viruliferous whiteflies coming from external sources of inoculum. Severe epidemics are recurrently observed in some tomato fields in several Brazilian regions, which prompted us to postulate the existence in the agroecosystem, in some places and time, of amplifier hosts that provide the necessary force of infection for epidemics to occur, even in the absence of secondary spread in the target crop. Amplifier hosts are ideally asymptomatic, occur in high density near the target crop, and support growth of both virus and vector. Soybean and common bean are potential amplifier hosts for begomovirus in tomato crops. Our results support the hypothesis that soybean plants may play an important role as an amplifier host of ToSRV for tomato crops in the field, although this does not seem to be a frequent phenomenon. Successful amplification will depend on several factors, including the soybean cultivar, the soybean stage of development at the moment of infection, the ToSRV isolate, and the perfect synchrony between the beginning of a soybean field and the end of a ToSRV-infected crop, and, later, between the senescence of the ToSRV-infected soybean plants and the new tomato crop. The concept of amplifier hosts has been widely used in ecology of zoonoses but, to our knowledge, has never been used in botanical epidemiology.

## Introduction

Tomatoes are one of the most important vegetable crops in Brazil and worldwide. In 2018, approximately 59.7 thousand hectares were planted to tomatoes in the country, and 4.1 million tons of fruit was produced (Instituto Brasileiro de Geografia e Estatística [IBGE], 2018). Tomato production can be affected by several begomoviruses, the most prevalent being tomato severe rugose virus (ToSRV) (Fernandes et al., 2008; Inoue-Nagata et al., 2016; Rojas et al., 2018; Mituti et al., 2019). Begomoviruses are transmitted by the silverleaf whitefly *Bemisia tabaci* in a circulative-persistent manner (Rosen et al., 2015).

In 2003, due to the high incidence of begomovirus in processing tomatoes, a legislative control measure mandating a tomato-free period of 2 months (December and January) was implemented in Goiás state (Inoue-Nagata et al., 2016). Despite the implementation of this legislative control measure, a high incidence of ToSRV (60 to 100%) is recurrently observed in tomato crops (Macedo et al., 2014, 2017c, 2019).

Current control of diseases caused by begomoviruses for processing (determinate growth) and fresh market tomatoes (indeterminate growth) depends almost exclusively on both planting resistant/tolerant hybrids and on intensive insecticide sprays (2 to 3 per week) for controlling *B. tabaci*, the vector of ToSRV, in the target crop. Resistant hybrids possess only moderate resistance to infection with the bipartite begomovirus ToSRV, as well as some tolerance to the disease (Inoue-Nagata et al., 2016). Insecticide sprays, although widely used, have failed in most tomato production areas in Brazil because they are unable to reduce primary spread, i.e., infection caused by the influx of viruliferous whiteflies coming from external sources of inoculum. This failure has been reported for a begomovirus disease in tomato fields in Florida (Polston et al., 1996) and demonstrated under controlled conditions by Gouvêa et al. (2017) in Brazil. As for processing tomatoes, high incidences of ToSRV in fresh market tomatoes are observed annually in some tomato fields in Brazil.

These recurrent and localized abrupt high incidences of ToSRV prompted us to propose a hypothesis for such epidemics: the existence in the agroecosystem, in some places and time, of amplifier hosts that provide the necessary force of infection for epidemics to occur, even in the absence of secondary spread in the target crop. Force of infection is defined as the rate at which susceptible target individuals acquire an infectious disease from a given source (Viana et al., 2014). Amplifier hosts are ideally asymptomatic, occur in high density near the target crop, and support the abundant growth of both virus and vector. The amplifier host acts as an intermediate link between a reservoir host and the target host and provide a strong short-term source of infection. The concept of amplifier hosts has been widely used in ecological studies of zoonoses (Childs et al., 2007; Lambin et al., 2010; Karesh et al., 2012; Jones et al., 2013; Streicker et al., 2013; Wardrop, 2016; Faust et al., 2018), but to our knowledge has never been used in botanical epidemiology.

## Reservoir and Amplifier Hosts in Botanical Epidemiology

The amplifier-host hypothesis applied to ToSRV epidemiology is based on the premise that secondary spread of the virus in tomato fields with high insecticide input is almost fully prevented by the nearly complete elimination of *B. tabaci* from the tomato fields. Therefore, epidemics, when they occur, are driven by the migration of viruliferous whiteflies from outside the target field (Barbosa et al., 2016; Bergamin Filho et al., 2016; Macedo et al., 2017c, 2019). This indicates the crucial importance of identifying the external sources of inoculum, as recognized recently in landscape botanical epidemiology (Plantegenest et al., 2007; Meentemeyer et al., 2012; Bergamin Filho et al., 2016).

Epidemiology of plant viruses has traditionally considered the reservoir as the main source of primary inoculum; afterward, the epidemic is driven by the secondary inoculum (Duffus, 1971). Reservoir is defined as one or more epidemiologically connected populations (mainly weed hosts in plant virus epidemics) in which the pathogen can be permanently maintained and from which infection is transmitted to the target population (Haydon et al., 2002). Reservoir hosts provide a long-term source of infection to the target population (Reisen, 2010). However, in Brazilian conditions, we have strong evidence that the incidence of ToSRV in populations of weed plants (reservoir) is very low (Macedo et al., 2017c; Rezende, unpublished data), and secondary spread is prevented by intensive and effective insecticide sprays for vector control.

**FIGURE 1:**
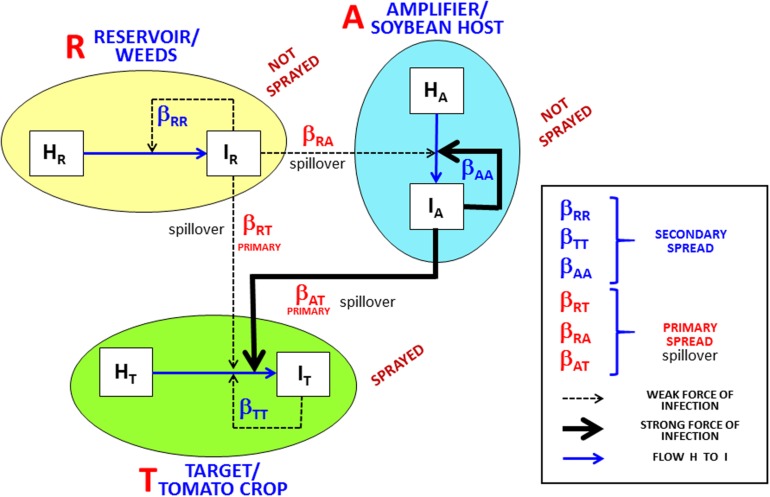
Framework of the target (tomato crop)/reservoir (weeds)/amplifier (soybean host) pathosystem. Target tomato crop (T) receives inoculum influx (spillover) from the reservoir (R), with a weak primary infection force (β_*RT*_) due to low infected reservoir density, and from the amplifier (A), with a strong infection force (β_*AT*_) due to high infected amplifier density. H and I represent healthy and infected plants, respectively, for target, reservoir, and amplifier. Rates β_*RR*_, β_*TT*_, and β_*AA*_ drive secondary infections in R, T, and A, respectively; thin dotted lines in R represent weak forces of secondary infection due to low infected plant density; thin dotted lines in T represent weak forces of secondary infection due to intensive insecticide sprays; thick line in A represents a strong force of secondary infection due to high density of infected plants and absence of insecticide spray. Spillover is defined as a primary infection from a host species to a different host species. Based on Fenton and Pedersen (2005) with addition of the amplifier host concept.

The amplifier-host hypothesis (Figure 1) helps to explain the recurrent rapid epidemics that occur in Brazilian conditions, despite both the weak reservoir force of infection and the prevention of secondary spread by insecticide sprays for vector control. The insight to include amplifier hosts in the conceptual model of ToSRV/tomato epidemics was based on two recent surveys carried out in central Brazil, one reporting that 2.9% of asymptomatic common bean plants were infected with ToSRV (Macedo et al., 2017a), and the other reporting that 3.3% of asymptomatic soybean plants were infected with the same begomovirus (Macedo et al., 2017b). Furthermore, data collected in 2018 in the Sumaré region (state of São Paulo) in a senescent soybean crop near a recently transplanted tomato crop (sprayed with insecticide three times per week) showed a > 10% incidence of asymptomatic ToSRV-infected soybean plants and a 57–70% incidence of symptomatic ToSRV-infected tomato plants. Infection of both the soybean and tomato plants with ToSRV was confirmed by means of PCR, using the degenerate primer pairs PAR1c496 and PAL1v1978 for begomoviruses (Rojas et al., 1993), followed by nucleotide sequencing of the amplicons. The approximately 300,000 soybean plants per hectare would provide 30,000 ToSRV-infected plants per hectare as sources of inoculum (amplifier). Tomato fields are often located next to common bean and soybean fields on Brazilian farms. The potential interplay of begomoviruses and these crops has been mentioned previously (Costa, 1975; Gilbertson et al., 1991; Navas-Castillo et al., 1999; Anderson et al., 2004).

## Soybean as an Amplifier Host to ToSRV Epidemics in Tomato

In order to obtain further information on the potential of soybean plants to act as an amplifier host, we studied the susceptibility of 42 soybean cultivars to ToSRV infection in three independent experiments performed under greenhouse conditions and in one experiment in the field. Healthy soybean plants were grown from seeds in 3.0-liter pots containing substrate. After reaching the 3 to 4 true-leaf stage, the plants were inoculated with ToSRV. Virus-free adults of *B. tabaci* MEAM1, reared on collard plants (*Brassica oleracea*) kept in whitefly-proof cages were provided an acquisition access period (AAP) of 24 h on ToSRV-infected tomato leaves. Afterward, these potentially viruliferous insects were transferred to five plants of each cultivar, growing in separate pots that were covered with voile fabric cages. An average of 30 insects per plant were released into the cage. The inoculation access period (IAP) was 96 h. Then, the plants were sprayed with the insecticide Flupyradifurone (Sivanto^®^) to prevent later colonization by the vector, and kept in whitefly-proof cages in a greenhouse. ToSRV was detected by PCR in total DNA extracted from soybean leaf samples according to Dellaporta et al. (1983), modified according to Favara et al. (2019). Samples of total DNA extracted from ToSRV-infected tomato plants and from healthy plants were used as positive and negative controls, respectively. The PCR was performed with the primer pairs PAR1c496 and PAL1v1978 (Rojas et al., 1993). The amplicons obtained were analyzed by electrophoresis in 1% agarose gels, stained with SYBR^®^ Safe DNA Gel Stain (Invitrogen), and visualized in a UV-transilluminator. Some amplicons were randomly selected, purified, and sent for nucleotide sequencing at Macrogen Inc. (Seoul, South Korea) to confirm the identity of the virus. In the first experiment, ToSRV was also detected by qPCR with the specific primers ToSRV-F 5′-GCAACCGCCTCTAGCACTTC-3′ and ToSRV-R 5′-GACCTGGTCTCCCCAACAAGG-3′ and protocols described by Bampi et al. (2019).

After evaluating the susceptibility of soybean cultivars to ToSRV infection under greenhouse conditions, an experiment was conducted in the field. First, 100 healthy tomato plants were transplanted in the field. Then, 10 ToSRV-infected *Nicandra physaloides*, were transplanted around the tomato plants to act as sources of inoculum. To ensure the presence of the vector, 4 collard plants infested with *B. tabaci* MEAM1 were placed in the field, inside whitefly proof-cages. Once a week the cages were opened to release some insects. The infection of the tomato plants with ToSRV was confirmed by observation of the symptoms and by PCR for randomly chosen plants. Sixty days later, when the rate of ToSRV-infected tomato plants was approximately 100%, soybean plants of 22 cultivars were exposed to natural infection with this virus in the field. The plants were grown from seed in 3.0-liter pots containing substrate. After emergence, ten plants of each cultivar were randomly placed in the field containing ToSRV-infected tomato plants. Ten healthy tomato plants cv. Santa Clara were also placed in the field as control of ToSRV transmission. Virus-free adults of *B. tabaci* MEAM1 were released 3 times (at 5, 10, and 15 days after plant exposure). Then, 40 days after exposing soybean plants to natural infection with ToSRV, PCR was performed with the primer pairs PAR1c496 and PAL1v1978 (Rojas et al., 1993).

Of the 42 soybean genotypes evaluated under greenhouse conditions, 14 were susceptible to infection with ToSRV (Table 1). The rate of infection ranged from 10% to 40%. Of the 22 cultivars evaluated under field conditions, the cultivars TMG 7262 RR, BRS 282, and BRS 284 were susceptible to ToSRV. The rate of infection ranged from 10% to 20%. The infection rate of tomato plants cv. Santa Clara was 50% (5/10). The susceptibility of soybean cultivars to ToSRV infection under experimental and field conditions varied. Only plants of cultivar TMG 7262 RR were infected under both conditions. All soybean-infected plants, as confirmed by PCR, were symptomless in both assays.

**TABLE 1:** Susceptibility of soybean cultivars to infection with tomato severe rugose virus experimentally inoculated using *Bemisia tabaci* MEAM1, and of cultivars exposed to natural infection in the field.

	**Greenhouse experiment^a^**		**Field experiment^b^**	
		
	**No. infected plants/No. inoculated**		**No. infected plants/No. inoculated**	
**Cultivar**	**plants**	**% Infection**	**plants**	**% Infection**
AFS 110	0/15	0	0/10	0
AMS Tibaggi Bayer	0/10	0	nt	nt
BMX Garra	2/10	20	nt	nt
BMX Ícone	2/10	20	nt	nt
BMX Potência	0/15	0	0/10	0
Bonus	0/10	0	nt	nt
BR 4	1/10	10	0/10	0
BR 16	1/10	10	nt	nt
BR 36	0/15	0	0/10	0
BR 132	0/10	0	nt	nt
BR 282	0/10	0	1/10	10
BR 284	0/10	0	1/10	10
BRS 245 RR	0/15	0	0/10	0
BS 2606 Ipro	0/10	0	nt	nt
Campos Gerais	2/15	13	nt	nt
CD 206	0/15	0	0/10	0
Davis	0/15	0	0/10	0
Desafio	0/10	0	nt	nt
Embrapa 48	0/15	0	0/10	0
FT Abyara	0/15	0	0/10	0
FT Cometa	0/15	0	0/10	0
FT-11 Alvorada	0/15	0	0/10	0
IAS3	0/15	0	0/10	0
M 6210 Monsoy	0/10	0	nt	nt
M 7251	0/10	0	nt	nt
M 5892 Ipro	1/10	10	0/10	0
M 72-S1	0/10	0	nt	nt
MG/BR 46 - Conquista	1/10	10	0/10	0
Nidera 5909	1/10	10	0/10	0
NS 6906 Ipro Nidera	0/10	0	nt	nt
NS 6909 Ipro Nidera	0/10	0	nt	nt
Ocepar 3 - Primavera	2/10	20	nt	nt
Ocepar 4 - Igua u	2/10	20	nt	nt
Ocepar 5	0/10	0	nt	nt
Paraná	0/15	0	0/10	0
Paraná Marrom	0/15	0	0/10	0
Sambaiba	0/10	0	nt	nt
Santa Rosa	0/15	0	0/10	0
TMG 7062	1/10	10	nt	nt
TMG 7262 RR	4/10	40	2/10	20
TMG 7739	1/10	10	nt	nt
Vi oja	1/10	10	0/10	0

*^*a*^Three independent experiments. ^*b*^One experiment. nt, not tested.*

ToSRV-infected soybean plants of the cultivars TMG 7262 RR, Ocepar 4-Iguaçu, Viçoja, MG/BR 46-Conquista and M 5892 Ipro were then evaluated as sources of inoculum for *B. tabaci* MEAM1, for subsequent transmission to 13 healthy tomato plants, using an average of 30 insects per plant. The AAP and IAP were the same as the ones described for the previous experiment. The rates of ToSRV transmission from infected plants of cultivars TMG 7262 RR and Ocepar 4-Iguaçu were 23% (3/13) and 15% (2/13), respectively. The virus was not transmitted from infected plants of cultivars Viçoja, MG/BR 46-Conquista and M5892 Ipro to tomato plants.

## Discussion

These results support the hypothesis that soybean plants may play an important role as an amplifier host of ToSRV for tomato crops in the field, although this does not seem to be a frequent phenomenon. Successful amplification will depend on several factors, including:

• the soybean cultivar, since not all of them are susceptible to ToSRV infection; even among susceptible cultivars, not all acted as a source of virus for transmission to tomato by *B. tabaci* MEAM1;
• the soybean stage of development at the moment of infection;
• the ToSRV isolate. Soybean plants of cv. Davis inoculated by biolistics and adults of *B. tabaci* MEAM1 were infected with the ToSRV isolate used by Macedo et al. (2017b). However, plants of the same cultivar were not infected with the ToSRV isolate used in the present study.
• a perfect synchrony must exist between the beginning of a soybean field and the end of a ToSRV-infected tomato crop, and later, between the senescence of the ToSRV-infected soybean plants and the new tomato crop.

The conceptual model proposed here opens up a number of research avenues with the potential to improve both our understanding of the tomato/ToSRV and other related pathosystems (referred as polycyclic diseases with continuous primary spread by Bergamin Filho et al., 2016), and our competence to formulate more rational and sustainable management strategies, especially for intensive agriculture, as follow:

• to investigate other potential amplifier hosts (in addition to soybean and common bean) of ToSRV;
• to quantify the epidemiological effect of different amplifier hosts on tomato crops;
• to determine the influence that the distance between the amplifier host and the tomato crop may have;
• to quantify the effect of vector control (chemical, genetic, biological, etc.) carried out in amplifier hosts on ToSRV incidence in tomato crops;
• to quantify the effect of the absence of the amplifier host (or planting a resistant variety of it) in the vicinity area where tomato is the main crop;
• to verify the influence of delay and/or coincidence of planting dates of tomato and amplifier hosts in the ToSRV epidemics in tomato;
• to assess the effect of a tomato-free-period (legislative control) when amplifier hosts are present in the area.

## Data Availability Statement

The datasets generated for this study are available on request to the corresponding author.

## Author Contributions

AB elaborated the concept of amplifier host for plant virus diseases. MM, GF, FO, and JR conducted field evaluations for amplifier host identification. DB and FO conducted greenhouse and field trials with soybean genotypes. GF, DB, and FO performed molecular analysis. All authors assisted in analyses of data and discussed the results, revised, and contributed to writing the manuscript. MM and DB drafted the manuscript.

## Conflict of Interest

The authors declare that the research was conducted in the absence of any commercial or financial relationships that could be construed as a potential conflict of interest.
